# Hand grip strength and all-cause mortality risk in individuals with decreased bone mass: a study from NHANES database

**DOI:** 10.3389/fmed.2024.1452811

**Published:** 2024-12-11

**Authors:** Hongdong Sun, Jiayi Liu, Ruirui Tan, Xiaomei Zhang, Xin Qian, Chenxi Qi, Wei Qi

**Affiliations:** ^1^Department of Acupuncture and Tuina, Changchun University of Chinese Medicine, Changchun, China; ^2^Shenzhen Hospital of Integrated Traditional Chinese and Western Medicine, Shenzhen, China; ^3^Department of Tuina, Shenzhen Hospital of Traditional Chinese Medicine, Shenzhen, China; ^4^Department of Traditional Chinese Medicine, Liaoning University of Traditional Chinese Medicine, Shenyang, China; ^5^Traditional Chinese Medicine Orthopedics and Traumatology Department, Shenzhen Baoan Authentic TCM Therapy Hospital, Shenzhen, China

**Keywords:** osteoporosis, mortality risk, grip strength, aging, NHANES

## Abstract

**Objective:**

Previous studies have demonstrated that grip strength is associated with various health outcomes, including osteoporosis. However, the impact of grip strength on long-term mortality risk among individuals with low bone mass remains unclear. This study aims to investigate the association between grip strength and the risk of all-cause mortality in the population with low bone mass.

**Methods:**

We included 1,343 cases of decreased bone mass from the NHANES database spanning 2013 to 2014. All-cause mortality data were ascertained through linkage with national death index records up to December 31, 2015. Analysis was conducted using the Cox proportional hazards regression model, and we assessed result reliability through various model adjustments and hierarchical analyses, Schoenfeld’s global and individual tests are utilized to estimate the time-varying covariance in the Cox proportional hazards regression model’s hypothesis.

**Results:**

Throughout an average follow-up period of 69.5 months, 148 deaths were documented. After adjusting for covariates, a significant association between grip strength and the risk of all-cause mortality was observed in individuals with decreased bone mass (HR = 0.9, 95% CI: 0.87–0.93, *p* < 0.001). Individuals with normal grip strength, compared to those with low grip strength, exhibited a 56% lower risk of all-cause mortality (HR = 0.44, 95% CI: 0.29–0.67, *p* < 0.001). Various models consistently demonstrated similar significant trends post-adjustment. Subgroup analysis revealed an interaction between grip strength and coronary heart disease (*p* < 0.05). Schoenfeld’s global and individual tests confirmed the reliability of the model (*p* > 0.05).

**Conclusion:**

Our findings indicate that low grip strength is associated with increased all-cause mortality risk in individuals with decreased bone mass. The inclusion of routine monitoring of grip strength in patients with osteopenia and the encouragement of maintaining or improving grip strength in this population may offer a novel approach to health management for these individuals.

## 1 Introduction

Osteoporosis is a metabolic bone disease strongly associated with age. As global populations age, the incidence of osteoporosis continues to rise annually, imposing significant burdens on society (1). Osteoporotic fractures represent its most severe complication and are closely linked to patient mortality (2). Effective prevention of these complications is crucial for enhancing osteoporosis management.

Handgrip strength, quantified using a dynamometer, is a crucial indicator of muscle strength (3) and demonstrates a close relationship with osteoporosis. Research has shown that handgrip strength is associated with lumbar (4) and distal radius bone mineral density (BMD) (5). Additionally, low handgrip strength in postmenopausal Japanese women correlates with an increased risk of site-specific fractures over 10–15 years (6). Furthermore, diminished grip strength has been linked to higher mortality rates following hip fractures (7).

Handgrip strength measurement offers objectivity and high repeatability, making it a valuable tool for health and disease prediction research. As a simple, reliable, and inexpensive evaluation tool, handgrip strength assessment has shown significant predictive and diagnostic value in conditions such as sarcopenia (8), osteoporosis, and osteoporotic fractures (9). Previous studies have demonstrated that grip strength can serve as a predictor of total and cardiovascular mortality (10), and may also have a potential predictive role in the risk of all-cause death in elderly women with reduced bone mass (11). Grip strength also plays a pivotal role in osteosarcopenia (12).

While grip strength shows promise as a prognostic indicator for several diseases and correlates closely with BMD, research exploring its impact on long-term outcomes in individuals with decreased bone mass remains limited. Hence, this study investigates the relationship between grip strength and long-term mortality risk among people with osteopenia, aiming to provide evidence-based insights for their health management.

## 2 Method

### 2.1 Data source

Data concerning individuals with osteopenia were sourced from the publicly available National Health and Nutrition Examination Survey (NHANES) database. Mortality data were obtained from publicly released datasets by the United States Centers for Disease Control and Prevention (CDC). The NHANES database, managed by the National Center for Health Statistics (NCHS), aims to gather comprehensive health data across all age demographics in the United States. The NHANES protocol underwent evaluation by the NCHS Research Ethics Review Board, ensuring that all participants provided informed consent. We extracted data from the survey year 2013–2014, including BMD measurements, demographic details (age, gender, race, marital status, family income), anthropometric measures (height, weight, BMI, waist circumference), and questionnaire responses covering smoking habits, diabetes history, cardiovascular disease history, stroke history, and blood pressure levels. Detailed descriptions and measurement methodologies for each variable are available on the NHANES website.

### 2.2 BMD and low bone mass

We collected BMD data from the total femur, femoral neck, trochanter, and intertrochanter regions for the period spanning 2013 to 2014. BMD measurements were conducted using dual-energy X-ray absorptiometry (DEXA). The data were acquired with the Hologic QDR-4500A fan-beam densitometer (Hologic, Inc., Bedford, Massachusetts) equipped with Apex 3.2 software. Prior to each measurement, the densitometer was calibrated to ensure accuracy.

Osteoporosis and osteopenia were characterized by low bone mass. The criteria for these conditions were established according to the World Health Organization (WHO) standards (13). Osteopenia was defined as BMD values ranging from 1 to 2.5 standard deviations (SD) below the mean of young adult male and female reference populations. Osteoporosis was defined as BMD values more than 2.5 SD below the mean of the young reference population. The threshold of BMD for osteopenia and osteoporosis was based on the study of Looker et al. (14). The specific BMD thresholds for males and females can be found in the original study.

### 2.3 Hand grip strength

Hand grip strength was assessed using a hand grip meter. Each hand was tested three times, and the maximum sum of these readings across both hands was recorded as the comprehensive grip strength. We used half of the comprehensive grip strength as our metric for grip strength data. Given the known disparity in grip strength between males and females, we analyzed and categorized grip strength separately for each gender. We adopt the definition of sarcopenia for the European population as defined by the European Working Group on Sarcopenia in Older Adults (EWGSOP) (15). According to this standard, low grip strength is considered when male grip strength is below 27 kg and female grip strength is below 16 kg.

### 2.4 Covariates

Through an analysis of previously published studies, we identified factors associated with grip strength, osteoporosis, or mortality risk. These include general demographic characteristics such as age, gender, race, education level, household income poverty ratio (16, 17), marital status (18), body measurements including height (cm), weight (kg), and BMI (<25, 25–30, ≥30) (19). Additionally, we considered blood pressure (mmHg), past medical history including diabetes (yes/no), cardiovascular diseases: congestive heart failure (yes/no), coronary heart disease (yes/no), angina pectoris (yes/no), heart attack (yes/no), stroke (yes/no) and fracture (yes/no) (20, 21).

### 2.5 Sources of death data

The death data originates from the CDC and is linked to the NHANES database using a specific Inclusion Number. Further details regarding data conversions and links are available on the corresponding website.

### 2.6 Statistical analysis

Count data were presented as mean ± standard deviation, while measurement data were expressed as percentages. The analysis utilized a Cox regression model to examine the relationship between grip strength and mortality risk through both univariate and multivariate regression analyses. To assess result robustness, various models were constructed by adjusting covariates. Model 0 remained unadjusted. Model 1 incorporates general demographic characteristics including gender, age, race, marital status, family income, and education level. Model 2 builds upon Model 1 by adjusting for BMI and blood pressure. Model 3 further adjusts for smoking based on Model 2. Model 4 incorporates factors potentially increasing mortality risk, such as congestive heart failure, coronary heart disease, angina pectoris, myocardial infarction, and stroke. To handle missing data, we applied multiple imputation (MI) with 5 replications using the chained equation approach via the R mice package, enhancing statistical power and reducing bias. Five sets of complete data were generated, and their effect values were integrated. Model 5 represents the post-MI integrated effect values based on the fully adjusted model. Interaction and stratified analyses were conducted using subgroup variables. Schoenfeld’s global and individual tests were applied to assess the time-varying covariances in the Cox proportional hazards regression analysis.

All analyses were performed using R version 4.22 and FreeStatisticsV1.9.2 software. A significance level of *p* < 0.05 was applied for determining statistical significance.

## 3 Results

### 3.1 Baseline characteristics

The demographic profile of the study population is depicted in Table 1. A total of 1,343 individuals with decreased bone mass were enrolled, comprising 54.1% women. The mean age of the cohort was 62.0 ± 11.8 years. Among them, 137 participants exhibited low grip strength, while 1,206 had normal grip strength. By the end of 2015, 148 patients had deceased, with an average follow-up duration of 69.5 ± 13.9 months.

**Table 1:** Basic characteristics of included data.

	Total (*n* = 1,343)	Low grip strength (Male < 27 Kg Female < 16 Kg) (*n* = 137)	Non-low grip strength (Male ≥ 27 Kg Female ≥ 16 Kg) (*n* = 1,206)	*p*
Gender, *n* (%)				0.865
Male	617 (45.9)	62 (45.3)	555 (46)	
Female	726 (54.1)	75 (54.7)	651 (54)	
Age	62.0 ± 11.8	71.4 ± 10.1	60.9 ± 11.5	<0.001
Race, *n* (%)				0.508
Mexican American	152 (11.3)	20 (14.6)	132 (10.9)	
Other Hispanic	110 (8.2)	10 (7.3)	100 (8.3)	
Non-Hispanic White	711 (52.9)	65 (47.4)	646 (53.6)	
Non-Hispanic Black	171 (12.7)	21 (15.3)	150 (12.4)	
Other Race	199 (14.8)	21 (15.3)	178 (14.8)	
Education, *n* (%)				0.009
Less than high school	295 (22.0)	37 (27)	258 (21.4)	
High school diploma	296 (22.0)	40 (29.2)	256 (21.2)	
More than high school	752 (56.0)	60 (43.8)	692 (57.4)	
Marital status *n* (%)				0.018
Married	788 (58.7)	65 (47.4)	723 (60)	
Widowed/Divorced/Separated	447 (33.3)	59 (43.1)	388 (32.2)	
Never married	108 (8.0)	13 (9.5)	95 (7.9)	
Household income poverty ratio, *n* (%)				<0.001
≤1	237 (19.3)	28 (22.8)	209 (18.9)	
1< to ≤3	481 (39.2)	66 (53.7)	415 (37.6)	
>3	508 (41.4)	29 (23.6)	479 (43.4)	
Weight (kg)	73.3 ± 16.4	68.4 ± 13.9	73.8 ± 16.6	<0.001
Height (cm)	164.9 ± 10.0	160.4 ± 9.7	165.5 ± 9.9	<0.001
BMI	26.9 ± 5.2	26.7 ± 5.6	26.9 ± 5.2	0.686
Diabetes, *n* (%)				0.001
Yes	208 (15.5)	34 (24.8)	174 (14.4)	
No	1,135 (84.5)	103 (75.2)	1,032 (85.6)	
Smoking, *n* (%)				0.912
Yes	653 (48.6)	66 (48.2)	587 (48.7)	
No	690 (51.4)	71 (51.8)	619 (51.3)	
Congestive heart failure, *n* (%)				0.008
Yes	52 (3.9)	11 (8)	41 (3.4)	
No	1,291 (96.1)	126 (92)	1,165 (96.6)	
Coronary heart disease, *n* (%)				0.001
Yes	89 (6.6)	18 (13.1)	71 (5.9)	
No	1,254 (93.4)	119 (86.9)	1,135 (94.1)	
Angina pectoris, *n* (%)				0.08
Yes	59 (4.4)	10 (7.3)	49 (4.1)	
No	1,284 (95.6)	127 (92.7)	1,157 (95.9)	
Heart attack, *n* (%)				<0.001
Yes	77 (5.7)	17 (12.4)	60 (5)	
No	1,266 (94.3)	120 (87.6)	1,146 (95)	
Stroke, *n* (%)				<0.001
Yes	72 (5.4)	26 (19)	46 (3.8)	
No	1,271 (94.6)	111 (81)	1,160 (96.2)	
SBP (mmHg)	127.3 ± 18.7	133.2 ± 24.0	126.6 ± 17.9	<0.001
DBP (mmHg)	69.4 ± 13.4	64.9 ± 16.6	69.9 ± 12.9	<0.001
Status, *n* (%)				<0.001
Alive	1,195 (89.0)	86 (62.8)	1,109 (92)	
Deceased	148 (11.0)	51 (37.2)	97 (8)	
Follow-up time (month)	69.5 ± 13.9	58.9 ± 22.5	70.7 ± 12.0	<0.001
Fracture, *n* (%)				0.225
Yes	476 (35.4)	55 (40.1)	421 (34.9)	
No	867 (64.6)	82 (59.9)	785 (65.1)	
BMD, *n* (%)				<0.001
Osteopenia	1,185 (88.2)	98 (71.5)	1,087 (90.1)	
Osteoporosis	158 (11.8)	39 (28.5)	119 (9.9)	

Analysis of baseline data revealed that individuals with higher grip strength were predominantly younger, more educated, and had a higher weight and height. Additionally, this subgroup exhibited lower prevalence of cardiovascular and cerebrovascular diseases.

### 3.2 Association between grip strength and mortality

**Figure 1:**
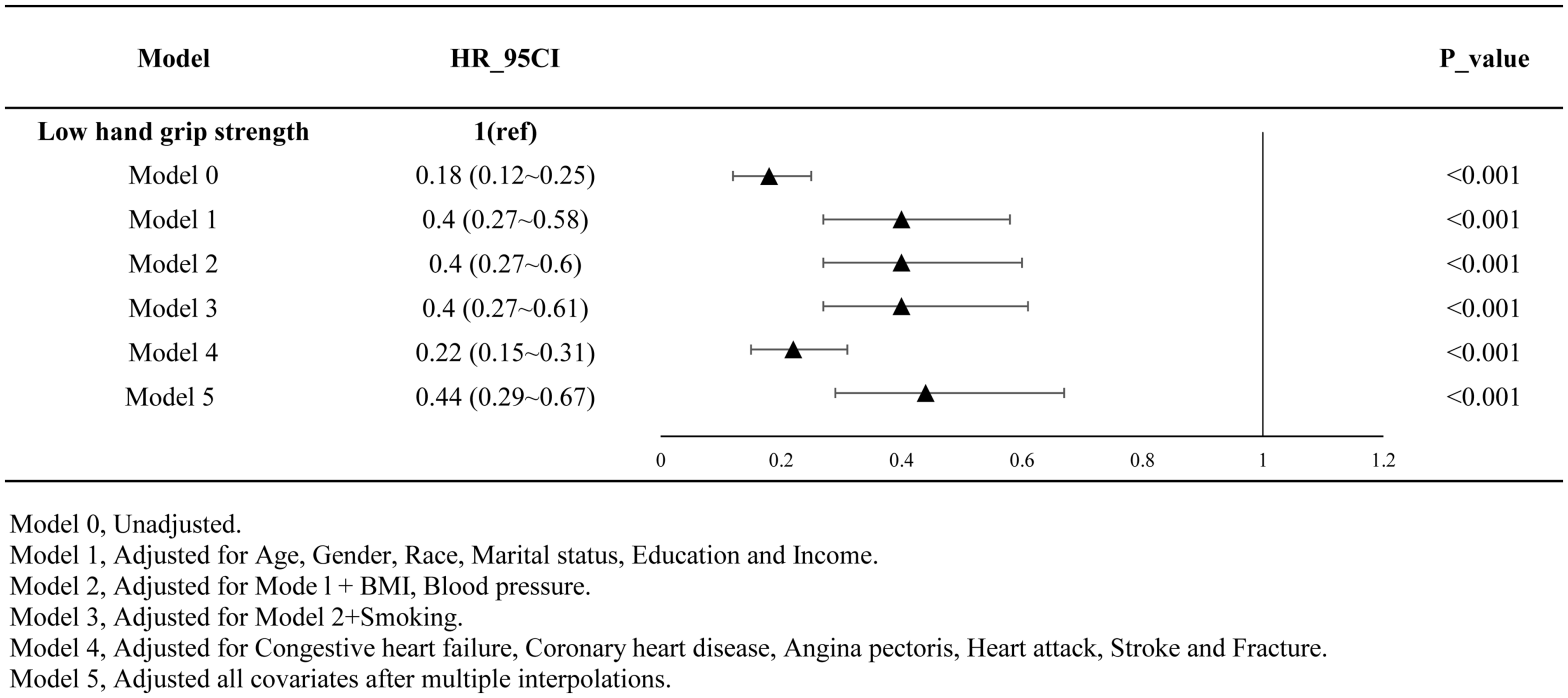
Results after multi-model adjustment (adjusted).

Table 2 presents the results of multivariate regression analysis investigating the association between grip strength and the risk of all-cause mortality among individuals with decreased bone mass. The analysis revealed a gradual reduction in the risk of mortality with increasing grip strength as a continuous variable (HR = 0.90, 95% CI: 0.87–0.93, *p* < 0.001). When comparing individuals with low grip strength to those with non-low grip strength, the latter exhibited a 56% lower risk of all-cause mortality (HR = 0.44, 95% CI: 0.29–0.67, *p* < 0.001). This trend remained consistent across various adjustment models, as depicted in Figure 1.

**Table 2:** Cox regression analysis results between grip strength and risk of all-cause mortality after adjusting for covariates.

	n.total	n.event (%)	Follow up time (month)	HR_95CI	*P*_value
Hand strength (kg)	1,343	148 (11)	93,277	0.9 (0.87 ~ 0.93)	<0.001
Low grip strength	137	51 (37.2)	8,067	1(Ref)	
Non-low grip strength	1,206	97 (8)	85,210	0.44 (0.29 ~ 0.67)	<0.001

### 3.3 Subgroup analysis

**Figure 2:**
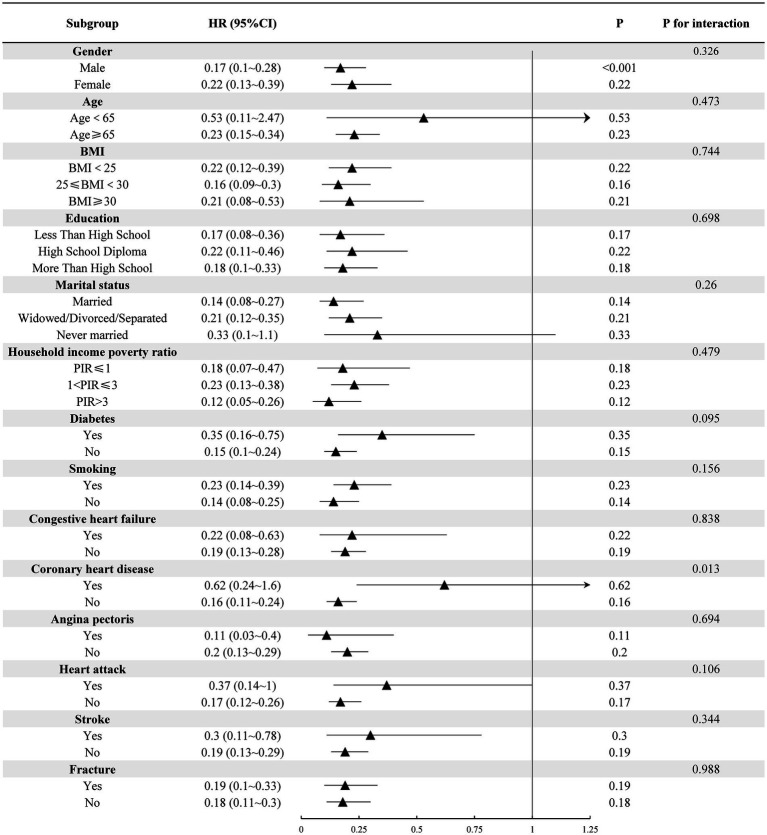
Subgroup analysis of hand grip strength and all-cause mortality risk in individuals with decreased bone mass.

Figure 2 displays the results of our subgroup analysis. Subgroup analysis results indicated that, although no significant difference was observed, the negative correlation trend of high grip strength on the risk of all-cause mortality among each subgroup remained consistent with the main analysis. Notably, a history of coronary heart disease exhibited a significant interaction with grip strength and the risk of all-cause mortality (*p* < 0.05).

### 3.4 Cox proportional hazard assumption test

**Figure 3:**
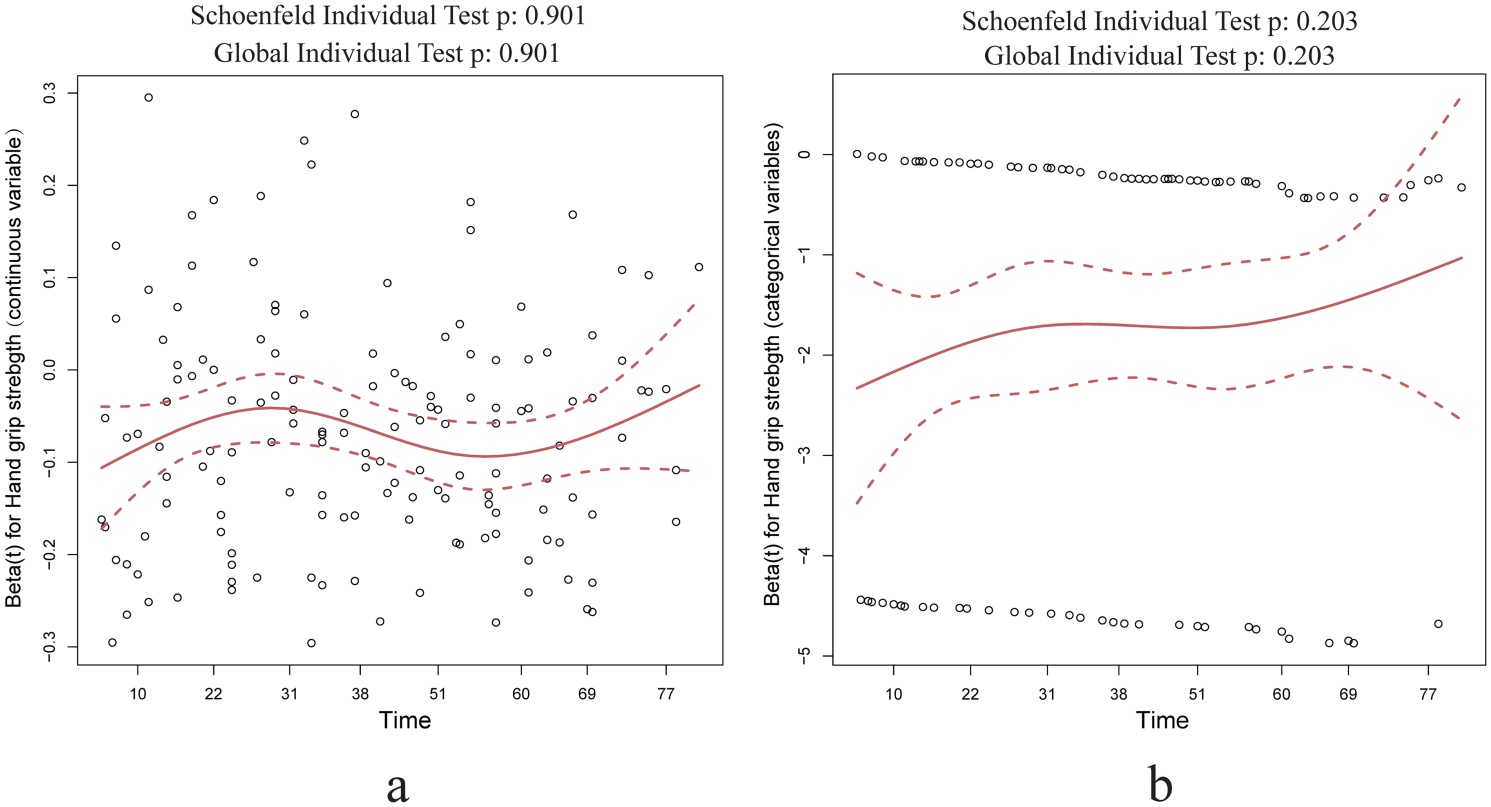
Schoenfeld residual test results of hand grip strength in Cox regression analysis. **(a)** Hand grip strength as a continuous variable Schoenfeld residual test results; **(b)** hand grip strength as a categorical variable Schoenfeld residual test results.

According to the Cox proportional hazards regression model, the hazard ratio of individual variables, such as hand grip strength, remains constant over time. We conducted a Cox proportional hazards model test using Schoenfeld residuals to evaluate the continuous and categorical variables of grip strength, respectively. The results, depicted in Figure 3, demonstrate that Schoenfeld’s individual and global tests do not suggest a violation of the proportional hazards assumption (*p* > 0.05).

## 4 Discussion

Our findings indicate that low grip strength may elevate the risk of all-cause mortality in individuals with decreased bone mass. Specifically, compared to those with higher grip strength, individuals with low grip strength experience a 56% higher risk of all-cause mortality. This trend persists across adjustments in our model and in stratified analyses. Our sensitivity analysis also verifies the reliability of the model.

The association between grip strength and health outcomes has been extensively investigated. Previous studies have consistently linked lower grip strength to increased risks of all-cause mortality, cardiovascular mortality, myocardial infarction, and stroke (22). Additionally, grip strength has been associated with heightened risks of all-cause dementia and mortality (23). Studies have also demonstrated poorer outcomes in patients with type 2 diabetes who exhibit lower grip strength, suggesting the inclusion of grip strength monitoring in their health management (24).

Varying perspectives exist on the influence of grip strength on the risk of all-cause mortality among individuals with decreased bone mass. A study reported that decreased bone mass in the distal forearm is associated with an elevated risk of all-cause death, and this association remains unaffected by high grip strength (25). Conversely, a cohort study involving 909 participants in the UK (26) revealed that low grip strength significantly elevates the risk of cardiovascular and all-cause mortality, whereas femoral neck BMD does not correlate with any risk of death. Another investigation, involving 1,032 subjects, indicated that those with muscle weakness or osteopenia face considerably higher mortality risks than those without these conditions (27). Additionally, individuals with reduced muscle mass exhibited notably higher mortality risks. However, the coexistence of osteopenia and osteodystrophy did not significantly augment fracture or mortality risks beyond those linked to each condition independently, emphasizing the distinctions between osteopenia, sarcopenia, and hypodynamia. In contrast, a prospective cohort study of elderly women (11) demonstrated that the potential osteosarcopenia group exhibited a heightened risk of 10-year hip fracture and mortality compared to the normal group or those with isolated low bone mass, aligning with our findings. Nonetheless, our study presents divergent conclusions from previous research, potentially attributed to variations in definitions of decreased bone mass, sample sizes, and adjustments for confounding variables. The discordant conclusions on this subject may be influenced by several factors, including the lack of unified criteria for defining osteomyopenia, differences in BMD measurement sites, sample size, follow-up duration, data censoring, and other variables. Given the pivotal role of grip strength in predicting adverse risk outcomes and the absence of a consensus on this matter, continued investigation into this topic is necessary.

The impact of grip strength on the risk of all-cause mortality in individuals with decreased bone mass primarily arises from reduced muscle strength, increasing the risk of fractures—a relationship well-documented in previous studies (28–31). Fractures, particularly hip fractures, are significantly linked to elevated mortality risk (32). Additionally, grip strength is viewed as an indicator of nutritional status, with higher grip strength correlating with better physical function (33). Lower grip strength often indicates a higher prevalence of chronic diseases, thereby diminishing the body’s resilience to adverse outcomes.

Our study observed the interaction of coronary heart disease in the effect of grip strength on the risk of all-cause death in people with low bone mass. The relationship between CHD and grip strength is bidirectional. Grip strength has demonstrated significant predictive value for the risk of CHD and associated mortality (34–36). Conversely, patients with CHD often exhibit a tendency to reduce grip strength. A prospective Finnish study (37) with a 22-year follow-up period reported a marked decrease in hand grip strength among CHD patients, corroborated by another study (38), indicating that the risk escalates over the course of the disease. CHD is a pivotal determinant of all-cause mortality in patients, a topic extensively covered in previous research. Furthermore, decreased grip strength in CHD patients may exacerbate the risk of all-cause death. However, the specific impact of CHD on grip strength in osteopenia populations remains underexplored and warrants further investigation. Several limitations exist within this study. First, to maintain data integrity, cases with missing information were excluded, thereby reducing the number of included cases to some extent. Second, as the study primarily involves the American population, the generalization of results to other populations remains uncertain. Third, due to its observational design, causal associations cannot be established, and residual confounding factors may not be entirely excluded. Fourth, without weight analysis, the findings may not fully represent the characteristics of the entire NHANES survey population. Finally, the number of deaths involved is relatively small compared to the sample size, which may limit the statistical power of the study. Further verification through studies with larger sample sizes is necessary.

## 5 Conclusion

Low grip strength is associated with an increased risk of all-course mortality among individuals with decreased bone mass. Integrating routine monitoring of grip strength in patients with osteopenia and promoting the maintenance or enhancement of grip strength in this population may introduce a novel strategy for health management among these individuals.

## Data Availability

The original contributions presented in the study are included in the article/supplementary material, further inquiries can be directed to the corresponding author.
